# A Sensor-Data-Driven Proactive Accident Detection and Traffic Prediction Method Based on Lane-Level Grid Partitioning and a Three-Dimensional Markov Model

**DOI:** 10.3390/s26175594

**Published:** 2026-09-03

**Authors:** Meng Zeng, Hang Chen

**Affiliations:** College of Engineering, Zhejiang Normal University, No. 688 Yingbin Road, Jinhua 321001, China; chenhang@zjnu.edu.cn

**Keywords:** sensor data, traffic accident, traffic condition, rectangular grid, Markov model

## Abstract

**Highlights:**

**What are the main findings?**

- The proposed sensor-driven method achieves lane-level accident detection and traffic prediction with high accuracy by fusing historical and real-time data within a three-dimensional Markov model.
- The proactive detection mechanism substantially shortens detection latency, reducing secondary accident risks and congestion propagation.

**What are the implications of the main findings?**

- The framework offers a practical sensor-enabled solution for real-time traffic surveillance and intelligent transportation management in big-data environments.
- The method is also applicable to diagnosing congestion and other traffic anomalies, supporting comprehensive roadway incident monitoring and proactive control strategies.
- A sensor-data-driven proactive accident detection and traffic prediction method based on lane-level grid partitioning and a three-dimensional Markov model.

**Abstract:**

The timely detection of road traffic accidents is essential for intelligent transportation systems. Leveraging multi-source sensor data including GPS, loop detectors, and vehicular sensors, this study proposes a proactive accident diagnostic method within a big-data framework. We introduce a lane-level traffic state representation that discretizes each lane into rectangular grids, enabling precise evaluation of local traffic conditions. To capture the spatio-temporal propagation of traffic disturbances, a three-dimensional Markov model is adopted, which accounts for both upstream–downstream traffic spread and temporal evolution, as well as historical features, to predict post-accident traffic dynamics. Experimental results demonstrate that the proposed method achieves high-accuracy lane-level accident detection and improves traffic prediction performance through the effective fusion of historical sensor records with real-time streaming data. The proactive detection mechanism efficiently reduces accident identification time, thereby mitigating potential secondary impacts. Additionally, the method proves effective in diagnosing other traffic anomalies, such as congestion, and for continuous monitoring of roadway incidents. These findings provide a practical sensor-enabled solution for accident detection and traffic flow prediction, offering a robust basis for real-time traffic management under intelligent network and big-data environments.

## 1. Introduction

With the rapid growth of motor vehicles, urban congestion has become increasingly severe. Concurrently, the frequency of traffic accidents has risen substantially, resulting not only in casualties and property damage but also in traffic jams and even secondary accidents at the incident sites. Therefore, an efficient accident detection strategy is crucial for traffic management. Faster detection enables quicker responses, which reduces the handling time, alleviates traffic pressure, and lowers the likelihood of secondary accidents.

Currently, accident reporting largely depends on telephone calls or alarm systems. Accidents occurring within areas covered by monitoring devices can be detected promptly. However, in locations without such equipment, accident notification remains delayed. Such delays can aggravate traffic pressure, lead to congestion, and even trigger secondary accidents. Hence, timely awareness of an accident is a key factor in mitigating its negative impacts.

As an outcome of intelligent transportation systems (ITS), advanced data collection technologies, such as roadside sensors and in-vehicle devices, offer a new data-driven means of capturing traffic motion characteristics. By detecting changes in these characteristics, abnormal conditions can be identified in a timely manner. This proactive detection approach enables traffic managers to monitor accidents effectively. Moreover, tracking variations in traffic states provides a solid foundation for diagnosing traffic disorders and congestion. Nevertheless, most existing data-driven traffic-state-detection methods operate at the macroscopic level [1,2,3], i.e., analyzing traffic conditions at the road-section or corridor scale and therefore cannot pinpoint the exact lane where an incident occurs. This lack of lane-level granularity significantly limits the practical value of these methods for incident response. Moreover, these methods are predominantly reactive, which means they detect accidents only after they have been reported or after congestion has fully developed, rather than proactively identifying them from real-time traffic anomalies. These limitations inherently introduce detection delays and reduce the precision of incident management.

Beyond timely detection, forecasting post-accident traffic evolution is equally critical for effective accident managements. Such predictions enable traffic control centers to implement timely diversion strategies, dispatch emergency services more effectively, and reduce the risk of secondary accidents. However, existing studies on post-accident traffic prediction [4,5,6,7,8,9,10,11,12] have largely focused on either the temporal dimension or the spatial dimension, but rarely both simultaneously. This fragmented treatment overlooks the inherently coupled nature of traffic dynamics, i.e., disturbances propagate not only over time but also across adjacent lanes and along upstream–downstream directions.

**Figure 1:**
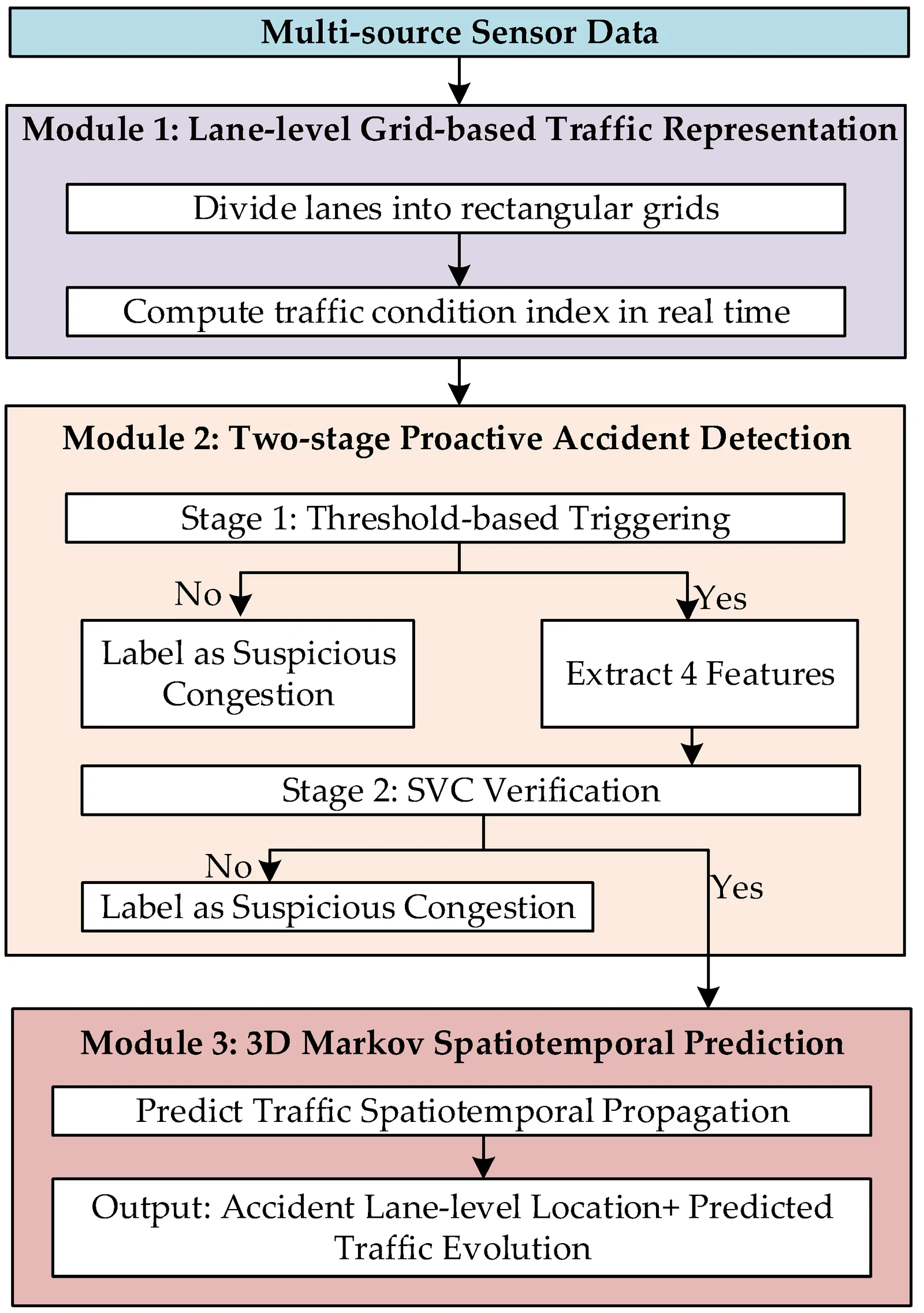
The framework of the proposed method.

To address the aforementioned issues, this paper proposes a lane-level proactive accident detection and prediction framework that fuses multi-source sensing data available in the era of big data and autonomous driving, including vehicle navigation systems, mobile phone data, in-vehicle sensors, and vehicle-to-road communication technologies. As illustrated in Figure 1, the framework discretizes each lane into rectangular grids and computes the length occupancy of each grid in real time from aggregated vehicle locations, enabling precise anomaly localization beyond the coarse road-section scale. For proactive identification, a two-stage mechanism combines threshold-based triggering with SVC verification, fusing historical and real-time data to distinguish actual accidents from routine congestion. Once an accident is confirmed, a three-dimensional Markov model captures the spatio-temporal propagation of traffic disturbances along upstream-downstream directions and over time. By evaluating the state of each grid, the framework enables timely accident localization and supports incident response and traffic diversion.

The contributions of this paper are as follows:

(1) Granularity contribution: We propose a lane-level grid-based traffic state representation that enables precise accident localization using multi-source sensing data, addressing the granularity gap of macroscopic methods.
(2) Proactivity contribution: We develop a two-stage proactive detection mechanism (threshold-based triggering + SVC verification) that fuses historical and real-time data to reduce detection latency and false alarms.
(3) Spatio-temporal prediction contribution: We establish a three-dimensional Markov model that captures the spatio-temporal propagation of traffic disturbances along upstream–downstream directions and over time, addressing the fragmented treatment of time and space in existing prediction approaches. Together, these contributions provide a sensor-enabled data-driven framework for real-time traffic surveillance and incident management.

The remainder of this paper is organized as follows. Section 2 reviews the related literature. Section 3 describes the method for traffic state determination (Module 1) and accident diagnosis (Module 2). Section 4 presents the prediction model for post-accident traffic conditions (Module 3). Section 5 presents the case study. This paper is concluded with Section 6.

## 2. Literature Review

Accident detection plays a critical role in transportation management, and the development of effective detection methods has attracted growing research attention over the past decades [13,14,15,16,17]. Most existing accident detection approaches rely on data collected from various traffic equipment [12,18,19], such as probe vehicles equipped with onboard GPS, traffic light systems, and surveillance cameras. Once an accident is detected, machine learning methods are commonly employed as efficient tools for accident analysis [20,21]. For instance, Ref. [22] assessed the impact of accidents using a support vector machine (SVM) model based on post-accident traffic data in Florida. Ref. [23] developed a hybrid model combining partial least squares and neural networks for traffic incident detection using average speed and traffic flow data. Ref. [24] respectively applied a nearest neighbor model, a regression tree, and a feed-forward neural network for accident detection. Beyond real-time detection, many studies have focused on investigating accident types and predicting the resulting damage [25,26,27,28].

Previous studies have predominantly performed traffic condition monitoring and accident detection from a macroscopic region-level perspective, with limited analysis at the lane level [1,2,3]. This macro-level approach leads to inaccuracies in both accident detection and post-accident impact assessment compared to lane-level or vehicle-level traffic state investigation. Furthermore, although accident detection has been explored in specific scenarios such as intersections, detection across the entire road network remains under-explored.

To quantify traffic changes in response to accidents, researchers have commonly employed traffic flow models, such as the traffic wave model, to study post-accident traffic variations [4]. Others have adopted machine learning methods for traffic prediction, including Bayesian networks, SVM, and Markov models. Among these, the Markov model is one of the most popular approaches for predicting traffic conditions [5,6,7]. For example, Ref. [8] divided a day into multiple periods and predicted traffic conditions in each period using Markov chains. Ref. [9] combined Markov models with traffic wave models to measure traffic variations. Ref. [10] employed a Grey–Markov model to predict highway traffic variations based on historical data, achieving acceptable accuracy and reliability. Ref. [11] combined the Bayesian information criterion with a Hidden Markov Model (HMM) for traffic prediction, demonstrating superior performance over SVM and LSTM-RNN (Long Short-Term Memory-Recurrent Neural Network). However, these studies primarily focus on temporal prediction, i.e., forecasting traffic conditions at a given location based on its own historical data, without explicitly modeling how traffic disturbances propagate spatially along upstream–downstream directions. Nevertheless, traffic flow naturally propagates from upstream to downstream, meaning that upstream traffic conditions inherently influence the downstream flow. Incorporating this upstream–downstream propagation into downstream traffic prediction could significantly improve accuracy. Moreover, although these machine learning methods can detect accidents once the relevant data becomes available; they do not proactively identify accidents from real-time traffic anomalies; rather, they rely on post-incident data or predefined incident reports. This reactive paradigm inevitably introduces detection delays, which aggravate congestion and increase the risk of secondary accidents.

In parallel with the approaches above, deep learning methods have gained increasing attention for traffic prediction. Graph Neural Networks (GNNs) are particularly suitable for transportation networks due to their graph-structured nature, with roads as nodes and edges, with traffic propagating along the topology. Comprehensive surveys by [29] and [30] review GNN applications in intelligent transportation systems, covering traffic forecasting, demand prediction, and autonomous vehicles. Within this family, Spatio-Temporal Graph Neural Networks (STGNNs), such as STGCN (Spatio-Temporal Graph Convolutional Network) and ASTGCN (Adaptive Spatial–Temporal Graph Convolution Network), combine GNNs for spatial dependency with recurrent or convolutional networks for temporal dynamics [31,32,33,34]. More recently, transformer architectures have been adapted for traffic prediction, leveraging self-attention to capture long-range dependencies [35].

While these deep learning approaches have achieved state-of-the-art performance on benchmarks, they typically require substantial computational resources and large training datasets. Moreover, current studies typically explore traffic propagation at the network level without providing detailed spatio-temporal distribution information at the lane level. The joint modeling of spatio-temporal propagation along upstream–downstream directions and over time remains a critical gap.

To address these limitations, this paper proposes a proactive detection method that integrates lane-level traffic condition monitoring with spatio-temporal traffic flow prediction. Specifically, we first characterize traffic conditions at the single-lane level using a rectangular grid-based approach, where each lane is divided into grids, and the length occupancy of each grid is computed in real time. A threshold-based triggering condition is then applied as a coarse filter to activate the subsequent verification stage. Upon activation, four discriminative features are extracted and fed into a Support Vector Classifier (SVC) with an RBF kernel to discriminate actual accidents from routine congestion. Once an accident is confirmed, we establish a three-dimensional Markov model that accounts for the dynamic spread of traffic over spatial grids and time to predict post-accident traffic evolution. By evaluating the traffic state of each rectangular grid, we can infer and locate accidents in a timely manner, facilitating accident handling and traffic relief.

## 3. Lane-Level Grid-Based Traffic State Representation and Accident Detection

### 3.1. Definition of Rectangular Grid

In previous studies on traffic accident detection, macroscopic traffic flow models, such as the traffic wave theory, were commonly employed to describe traffic conditions. However, the road occupancy and traffic flow variations induced by accidents are inherently lane-level phenomena. Specifically, traffic propagation originates from individual vehicles, then extends to lanes, and eventually affects multi-lane sections in the event of an accident. Nevertheless, detecting accidents based on vehicle-level traffic state prediction requires data from all the involved vehicles, which entails substantial computational overhead. Moreover, such prediction becomes infeasible when vehicle information is missing. To align with the propagation mechanism of traffic flow and to overcome the computational inefficiency and data sparsity, this study proposes a rectangular grid-based method for representing traffic conditions at the lane level.

**Figure 2:**
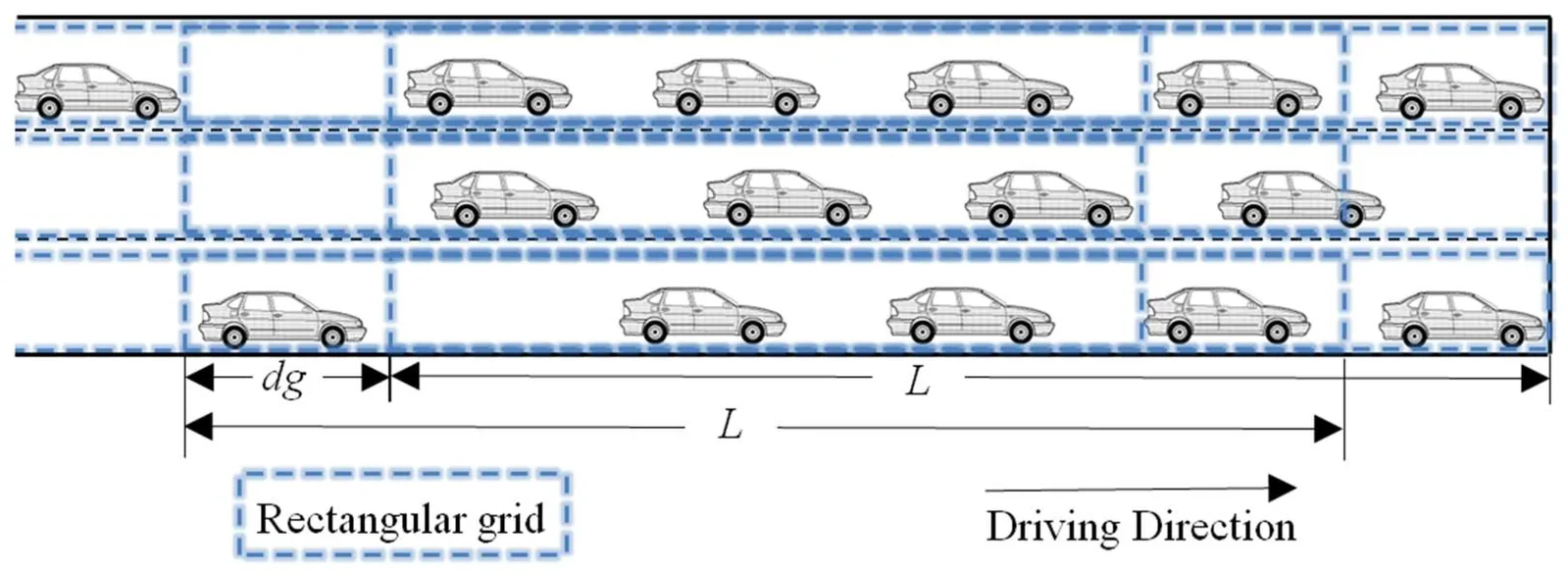
Illustration of rectangular grid.

As illustrated in Figure 2, each lane is divided into several rectangular grids with a unit length *L* and an inter-grid spacing *d_g_*. The unit length is determined based on the aggregation analysis of queuing vehicle density under different traffic conditions [36]. Specifically, *L* is defined as the sum of the lengths of five vehicles and the minimum safe gap (*d_min_*) among adjacent vehicles, where *d_min_* varies with driving scenarios. According to [37], *d_min_* is taken as 127.2 m for highway scenarios and 30.0 m for urban road scenarios. Assuming a uniform vehicle length of 5.0 m, the unit length *L* is calculated as 661.0 m for highways and 175.0 m for urban roads. The inter-grid spacing *d_g_* denotes the gap between two adjacent grids and is defined as the distance traveled by a vehicle in one second. Consequently, *d_g_* equals 33.3 m in highway scenarios and 10.0 m in urban road scenarios.

### 3.2. Traffic Condition Index: Length Occupancy

Based on the discretization of the single-lane traffic flow using rectangular grids, appropriate indicators must be selected to describe the traffic state within each grid and the variations between adjacent grids. According to [38], the occupancy parameter can effectively reflect the congestion and dissipation processes of road traffic flow and is widely used for traffic state estimation, particularly for local real-time applications. Therefore, this paper adopts occupancy as the descriptor for the traffic condition within each rectangular grid. Specifically, we employ the length occupancy, defined as the ratio of the sum of the projected lengths of all vehicles within a grid (in the direction of travel) to the unit length *L* of that grid. Correspondingly, the traffic flow speed can be inferred from the variation of traffic flow between grids. In the next section, where we predict traffic state evolution, we will describe the inter-grid traffic variation as the volume of traffic transmitted from the upstream grid to the downstream grid per unit time, thereby indirectly representing the traffic flow speed.

According to [39], the density and space occupancy exhibit a linear relationship under relatively stable traffic conditions, given by Equation (1).

(1) ρ=α∗R

where *R* denotes the space occupancy, α is a constant, and ρ is the traffic density. Hence, the number of vehicles *N* within a grid is

(2) N=L∗ρ=L∗α∗R

As previously stated, assuming a uniform vehicle length of 5.0 m, we define the length occupancy *μ* as the ratio of the total occupied length of all vehicles within a grid to the unit length *L* of that grid, as follows:

(3) $$\mu =\frac{N*l}{L}=\frac{L*\alpha *R*l}{L}=\alpha *R*l=\rho l$$

Let $\mu_{i,j}^{t}$ denote the length occupancy of the *i*-th grid in the *j*-th lane at time *t*. Accordingly, the matrix representing the length occupancy of all lanes at time *t* is given by

(4) $$\begin{array}{ccccc} \mu_{1,1}^{t} & \ldots & \mu_{i,1}^{t} & \ldots & \mu_{I,1}^{t}\\ \vdots & \vdots & \vdots & \vdots & \vdots \\ \mu_{1,j}^{t} & \ldots & \mu_{i,j}^{t} & \ldots & \mu_{I,j}^{t}\\ \vdots & \vdots & \vdots & \vdots & \vdots \\ \mu_{1,J}^{t} & \ldots & \mu_{i,J}^{t} & \ldots & \mu_{I,J}^{t} \end{array}$$

where in *J* is the total number of lanes, $j\in \left\{1,2,\ldots ,J\right\}$ denotes the lane index from the roadside, *I* is the number of grids per lane, and $i\in \left\{1,2,\ldots ,I\right\}$ is the grid index from the stop line at the intersection.

### 3.3. Two-Stage Proactive Accident Detection

#### 3.3.1. Threshold-Based Triggering Condition

As the prerequisite for traffic state classification, the length occupancy threshold needs to be determined. According to the basic traffic flow model:

(5) q=ρv,

we substitute Equation (3) into Equation (5) and get

(6) $$\mu =\frac{ql}{v}.$$

Therefore, when the traffic volume (*q*) is fixed, *μ* varies inversely with speed *v*. Hence, once *v* and *q* are determined, *μ* can be derived accordingly. Referring to the critical congestion values of *q* and *v*, the congestion threshold for occupancy is determined to be 0.28 [40].

**Figure 3:**
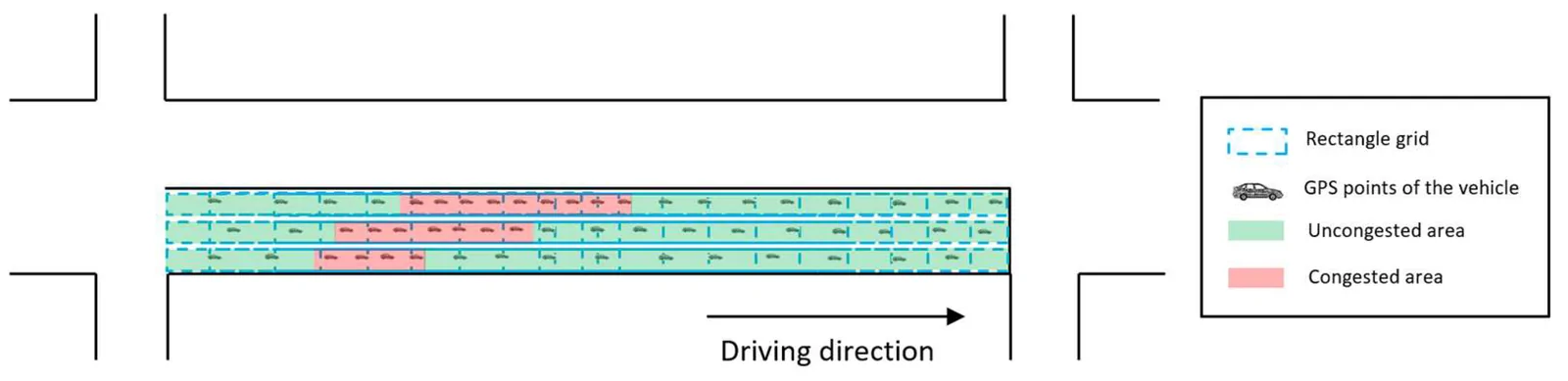
Lane-level traffic state recognition.

According to the obtained occupancy and delay values, the corresponding traffic states are then classified accordingly. This state information is subsequently incorporated into the spatio-temporal grid segments. Consequently, as illustrated in Figure 3, the real-time traffic state of each lane can be obtained.

When the length occupancy of two consecutive grids drops below 0.28, and the area remains congested for 30 consecutive seconds, the accident detection process will be activated.

#### 3.3.2. Feature Extraction and SVC-Based Verification

At this point, the historical state data of the corresponding grids must be retrieved. These data include daily-cycle information (i.e., the state at the same time of day over the previous seven days) and weekly-cycle information (i.e., the state at the same time on the same day of the week over the previous three weeks). Based on these data, a state evaluation model is established using the relative error as the performance metric.

Through the statistical analysis of traffic flow and accident data from the Seattle open database, four variables are identified as having the strongest correlation with accident discrimination: occupancy, state duration, daily-cycle same-time state (same-time state over the previous seven days), and weekly-cycle same-time state (the state at the same time on the same day of the week over the previous three weeks)). Accordingly, this study constructs a Support Vector Classifier (SVC) with a Radial Basis Function (RBF) kernel to determine whether an accident has occurred.

Let the training dataset consist of *K* samples, indexed by k=1,2,…,K, where each sample is represented by the feature vector $X_{k}={\left[\mu_{i,j}(t),\;t_{i,j}^{duration},\;\mu_{i,j}^{daily},\mu_{i,j}^{weekly}\right]}^{T}$ and its corresponding label $y_{k}\in \left\{+1,-1\right\}$, denoting whether an accident occurs (+1) or not (−1). $\mu_{i,j}(t)$ represents the current grid occupancy, $t_{i,j}^{duration}$ the duration of the current state at time *t*, $\mu_{i,j}^{daily}$ the daily-cycle same-time state, and $\mu_{i,j}^{weekly}$ the weekly-cycle same-time state. The SVC seeks an optimal separating hyperplane $W^{T}X+b=0$.

To handle the inherent nonlinearity and noise in traffic data, we adopt the soft-margin formulation with an RBF kernel:

(7) $$K(X_{k},X_{l})=exp(-\gamma {∥X_{k}-X_{l}∥}^{2})$$

where γ is the kernel width parameter, which controls the influence radius of each training sample and is determined via an exhaustive grid search over the range $\gamma \in \left[2^{-15},2^{3}\right]$ [41].

The model is solved via its dual form:

(8) $$\underset{\alpha }{\max}\sum_{k=1}^{K}\alpha_{k}-\frac{1}{2}\sum_{k=1}^{K}\sum_{l=1}^{K}\alpha_{k}\alpha_{l}y_{k}y_{k}K(X_{k},X_{l})$$

(9) $$such\;that\sum_{k=1}^{K}\alpha_{k}y_{k}=0,\;0\leq \alpha_{k}\leq C,\;k=1,2,\ldots ,K$$

where C is the penalty parameter, which balances the trade-off between maximizing the classification margin and minimizing the training error and is determined by an exhaustive grid search over the range $C\in \left[2^{-5},2^{15}\right]$.

Once the optimal Lagrange multipliers $\alpha_{k}$ are obtained, the final decision function for a new grid state X is given by

(10) $$f(X)=sign\left(\sum_{k=1}^{K}\alpha_{k}y_{k}K(X_{k},X_{l})+b\right)$$

If f(X)=+1, the system outputs an accident alert; otherwise, it is classified as a normal traffic state.

If a vehicle remains stationary for 30 consecutive seconds, the system identifies the occurrence of an accident at that vehicle’s location.

## 4. 3D Markov-Based Method for Spatio-Temporal Traffic Prediction

Accurate prediction of traffic flow under accident conditions is essential for effective accident management, as real-time awareness of the traffic state around the incident area facilitates rapid response and congestion mitigation. Referring to [42], the Markov model has demonstrated high accuracy in short-term traffic flow prediction using both historical and real-time data. Therefore, this paper employs a Markov model to forecast the evolution of traffic states after an accident occurs.

A Markov process characterizes the states of a system and the transition between different states. A Markov chain is a special case in which both time and state space are discrete. It satisfies the following two assumptions:

(1) The property of “without aftereffect “of the Markov model, which refers to the fact that the state $U^{t+1}$ of the system at time *t* + 1 depends only on the state at time *t* and is independent of all earlier states *t* − 1, *t* − 2, … That is, the value of $U^{t+1}$ depends only on the value of $U^{t}$ and not on the value of $U^{t-1}$, $U^{t-2}$, *…*.
(2) The state transition from time *t* to time *t* + 1 is independent of the state at time *t*.

In this paper, we define the system state at time *t* as the matrix comprising the length occupancy values of *I* grids across *J* lanes, denoted by $U^{t}$. Specifically,

(11) $$U^{t}=\left[\begin{array}{ccccc} \mu_{1,1}^{t} & \ldots & \mu_{i,1}^{t} & \ldots & \mu_{I,1}^{t}\\ \vdots & \vdots & \vdots & \vdots & \vdots \\ \mu_{1,j}^{t} & \ldots & \mu_{i,j}^{t} & \ldots & \mu_{I,j}^{t}\\ \vdots & \vdots & \vdots & \vdots & \vdots \\ \mu_{1,J}^{t} & \ldots & \mu_{i,J}^{t} & \ldots & \mu_{I,J}^{t} \end{array}\right]$$

where $\mu_{i,j}^{t}$ is the length occupancy of the *i*-th grid on the *j*-th lane at time *t*.

Generally, the traffic flow propagates from upstream to downstream. Consequently, the state of a grid is directly influenced by its upstream neighbor. Similarly, the state at time *t* + 1 depends on the state at time *t.* In addition, the historical data of a grid are also associated with its current traffic condition [42]. Hence, this paper establishes a 3D Markov model to predict traffic spatio-temporal propagation. The term “3D” refers to the three types of influence factors incorporated into the transition probabilities, namely, spatial (upstream–downstream), temporal (time step), and daily-cycle (same time on previous day).

(1) Influence of the upstream grid

Given the propagation of traffic states from upstream to downstream, a transition probability $P^{i+1\to i}$ is introduced to quantify the influence of the upstream grid on the downstream grid.

(12) $$P^{i+1\to i}=\frac{\mu_{i+1,j}^{t}}{\mu_{i,j}^{t}}$$

(2) Influence of the adjacent time step

Similarly, to capture the temporal dependency between consecutive states, we define the transition probability $P^{t-1\to t}$ from the state at time *t* − 1 to that at time *t* as follows:

(13) $$P^{t-1\to t}=\frac{\mu_{i,j}^{t-1}}{\mu_{i,j}^{t}}$$

(3) Influence of historical state

The grid’s state at time *t* on the previous day *d* − 1 is defined as $\mu_{i,j}^{t,d-1}$; the corresponding transition probability from the historical state to the current state at time *t* on day *d* is then calculated as

(14) $$P^{d-1\to d}=\frac{\mu_{i,j}^{t,d-1}}{\mu_{i,j}^{t}}$$

Considering all the three influencing factors, the compound transition probability is

(15) $$P_{i,j}^{t}=P^{i+1\to i^{\tau }}*P^{t-1\to t^{\varphi }}*P^{d-1\to d^{\omega }}$$

(16) such that  τ+φ+ω=1, τ, φ, ω≥0

where in τ, φ and  ω are influence intensities corresponding to the adjacent grid, the adjacent time step, and the historical state, respectively. By utilizing the MLE (maximum likelihood estimation) method [43], we obtain the values of τ, φ, and  ω as 0.43, 0.40, and 0.17, respectively, based on the data for ten days in one link of Yatai Street in Changchun city, China.

Substituting Equations (12)–(14) into (15), we get

(17) $$P_{i,j}^{t}={\left(\frac{\mu_{i+1,j}^{t}}{\mu_{i,j}^{t}}\right)}^{\tau }\cdot {\left(\frac{\mu_{i,j}^{t-1}}{\mu_{i,j}^{t}}\right)}^{\varphi }\cdot {\left(\frac{\mu_{i,j}^{t,d-1}}{\mu_{i,j}^{t}}\right)}^{\omega }=\frac{{\mu_{i+1,j}^{t}}^{\tau }\cdot {\mu_{i,j}^{t-1}}^{\varphi }\cdot {\mu_{i,j}^{t,d-1}}^{\omega }}{\mu_{i,j}^{t}}$$

Then, the transition probability matrix at time *t* is

(18) $$P^{t}=\left[\begin{array}{ccccc} P_{1,1}^{t} & \ldots & P_{i,1}^{t} & \ldots & P_{I,1}^{t}\\ \vdots & \vdots & \vdots & \vdots & \vdots \\ P_{1,j}^{t} & \ldots & P_{i,j}^{t} & \ldots & P_{I,j}^{t}\\ \vdots & \vdots & \vdots & \vdots & \vdots \\ P_{1,J}^{t} & \ldots & P_{i,J}^{t} & \ldots & P_{I,J}^{t} \end{array}\right]$$

The transition probability matrix at time *t* + 1 is

(19) $$U^{t+1}=U^{t}\cdot P^{t}$$

## 5. Case Study

### 5.1. Data Description

In this paper, we choose the underpass section of Qinggang Overpass on the northern expressway in Changchun as the case study section. The relevant traffic flow and accident data in December 2025 were provided by the traffic management department, of which the traffic flow data were mainly extracted from cameras and GPS data.

**Figure 4:**
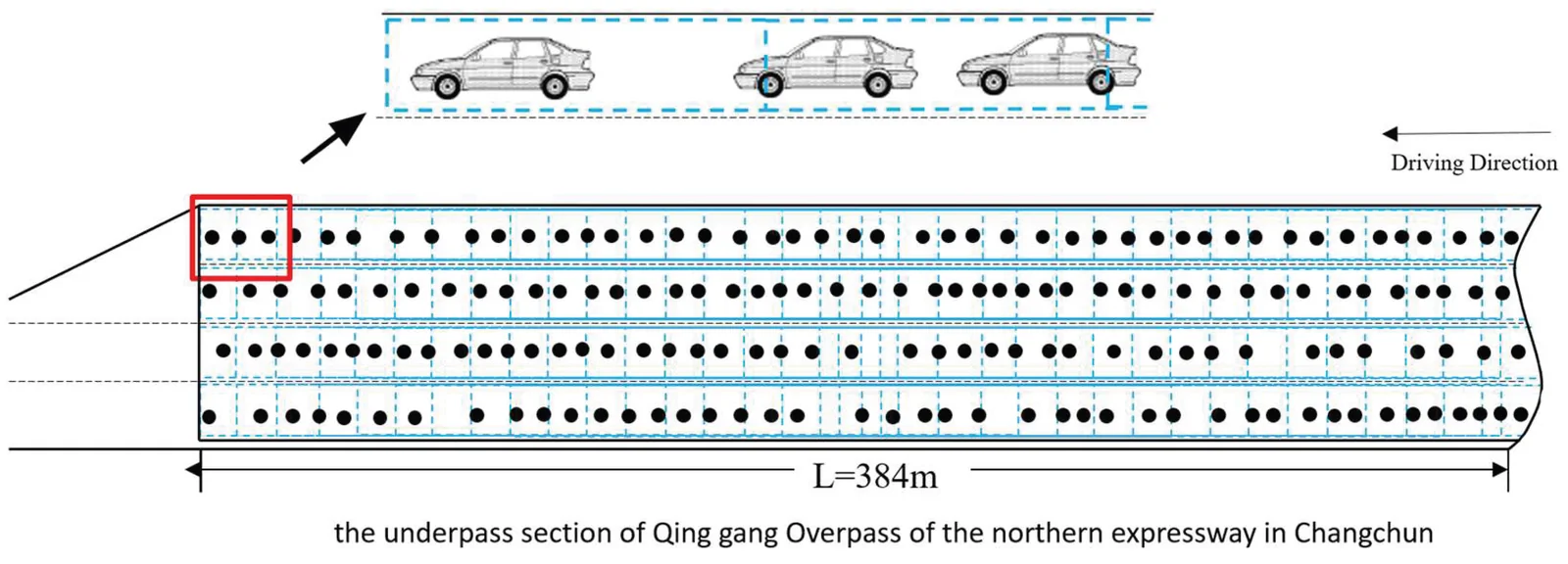
Rectangular grid illustration of the study section.

**Figure 5:**
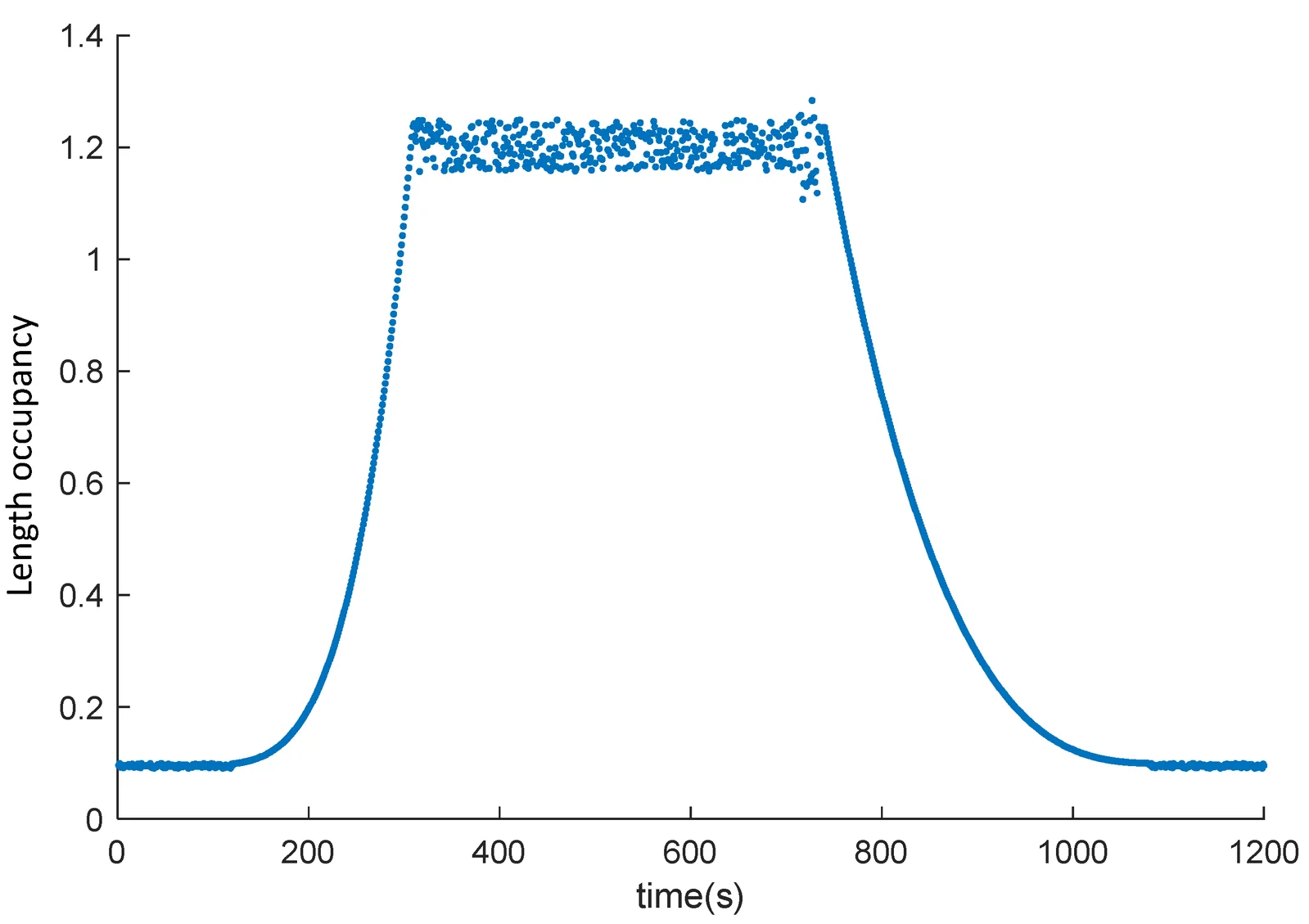
Traffic state of the accident spot.

As shown in Figure 4, the rectangular grid method is applied to discretize the road section into grids. On this road section, an accident occurred at 10:42:30 a.m. on 2 December 2025, providing the data for model verification. The traffic condition of the 384 m target section was first analyzed for the time window from 10:40 to 11:40. The results indicate that after 10:42:30, congestion developed in all lanes with varying occupancy levels. Notably, two vehicles exhibited zero speed in the 15th grid of the first lane during the interval from 10:42:30 to 10:59:28. The temporal evolution of that grid is presented in Figure 5.

As shown in Figure 5, the grid status undergoes four distinct phases: (1) uncongested from about 10:40 to 10:42; (2) extremely congested from about 10:42 to 11:01; (3) congestion evacuation from about 11:02 to 11:06; (4) free flow condition from 11:07 onward.

### 5.2. Traffic Prediction Performance

Using the historical data of 10:40–11:40 on 1 December 2025 and the current data at 10:40 on 2 December, we utilize the established Markov model to predict the post-accident traffic flow for the same time window (10:40–11:40) on 2 December. Across all grids and time steps, the proposed three-dimensional Markov model achieved an MAE of 0.020, an RMSE of 0.031, and a MAPE of 4.7%. The MAE of 0.020 indicates that, on average, the predicted occupancy deviates from the true value by only 2% of the maximum occupancy range. Notably, relatively large errors occur at the accident location and its immediate upstream grid, which can be attributed to the lack of accident records in the historical training data.

Moreover, to evaluate the robustness of the proposed Markov model to key parameter variations, we conducted sensitivity analyses for the influence weights (τ, φ, ω). These weights are determined as (0.40, 0.43, 0.17) by MLE under the constraint τ+φ+ω=1. To assess the contribution of each influence factor, we evaluated seven representative weight configurations, including the baseline, three single-factor configurations, three two-factor configurations, and an equal-weight baseline. Table 1 summarizes the results.

The results in Table 1 indicate that the baseline configuration of (τ = 0.40, φ = 0.43, ω = 0.17) achieves the best trade-off among the three dimensions. Specifically, the single-factor configuration performs poorly. The “Time only” configuration yields an MAE of 0.032, the “Spatial only” 0.045, and the “Daily only” 0.048, confirming that no single influence factor alone is sufficient for accurate traffic prediction.

Furthermore, two-factor configurations outperform single-factor ones. The “Time + Spatial” configuration achieves an MAE of 0.025, the “Time + Daily” configuration 0.030, and the “Spatial + Daily” configuration 0.041. This indicates that while any two factors are superior to one, incorporating all three factors is necessary to achieve optimal prediction accuracy.

It should also be noted that the equal-weight configuration yields an MAE of 0.026, which is 30% higher than the baseline. This confirms that simple uniform weighting is suboptimal. The baseline configuration assigns higher weights to the more influential factors (temporal and spatial propagation) and lower weight to the daily-cycle factor, which is consistent with the characteristics of traffic flow.

### 5.3. Accident Detection Performance Comparison

To evaluate the effectiveness of the proposed framework, as denoted in Table 2, we compared it against three baseline methods on the same dataset.

All methods were evaluated using the standard Automatic Incident Detection (AID) evaluation metrics: Detection Rate (DR), False Alarm Rate (FAR), Mean Time to Detect (MTTD), Precision, Recall, and F1-score [44]. The corresponding results are illustrated in Table 3.

The results in Table 3 reveal that the Threshold-only method achieves a high DR (95.0%) but with an extremely high FAR (45.2%). This confirms that the simple threshold condition alone is insufficient for reliable accident detection. It captures most actual accidents (high DR) but generates numerous false alarms under routine congestion such as peak-hour traffic, ramp merging, or signal-induced queuing. An FAR of 45.2% would render the system impractical in real-world deployment.

Moreover, comparing SVC (traditional) with the proposed method (both using SVC, but differing in feature sets), we observe that the addition of daily and weekly historical features substantially improves the performance. Specifically, the DR increases from 84.0% to 92.5%, the FAR drops from 14.5% to 4.2%, and the F1-score improves from 0.71 to 0.89. This demonstrates that historical pattern information ($\mu^{daily}$ and $\mu^{weekly}$) is critical for distinguishing accident-induced congestion from routine traffic variations. Without these features, the SVC can only rely on current *μ* and $t^{duration}$, which are insufficient to determine whether an observed congestion event is anomalous or historically expected.

In addition, compared with MLP (4 features), the proposed method outperforms the MLP across all metrics, achieving a higher DR (92.5% vs. 88.0%), a lower FAR (4.2% vs. 9.2%), and a shorter MTTD (28 s vs. 46 s). This indicates that for this specific classification task, the SVC’s margin-maximizing objective and kernel-based nonlinearity are more effective than the MLP’s feature learning, likely due to the limited size of the accident dataset (accidents are rare events) and the relatively low dimensionality of the feature space (4 features). In small-sample low-dimensional classification tasks, SVC tends to generalize better than neural networks.

Regarding the detection speed, the proposed method achieves the shortest MTTD (28 s) among all methods. There may be two factors contributing to this outcome. Unlike the threshold-only baseline, which relies on a fixed duration condition (≥30 s), the SVC-based verification operates as a streaming classifier that continuously evaluates features from the onset of congestion. In the evaluated accident cases, the SVC accumulated sufficient discriminative evidence, including sharp occupancy drops and deviations from historical patterns, and triggered an alert well before the 30 s fixed threshold was met. Additionally, compared to the SVC with only *μ* and $t^{duration}$ features, the proposed method with historical features ($\mu^{daily}$ and $\mu^{weekly}$) may detect anomaly signals earlier. Thus, the full feature set not only improves the accuracy but also accelerates detection.

Overall, the proposed method achieves a favorable balance between the detection sensitivity and false alarm control substantially, i.e., a DR of 92.5% and the lowest FAR (4.2%) among all methods, which is favorable for real-world deployment, as the false alarms can impose high operational costs and erode operator trust in the system.

### 5.4. Continuous Monitoring Performance

By continuously predicting the traffic states from December 1 to December 31, the model identified 85 congestion events and two accidents on the target section. The confusion matrix for the one-month detection period is presented in Table 4.

Specifically, for accident detection, the recall of 100% (2/(2 + 0) × 100% = 100%) indicates that no actual accidents were missed during the entire month. The precision of 33.3% (2/(2 + 4) × 100% = 33.3%) reflects the inherent rarity of accidents, only 2 out of 87 events were actual accidents, but the low false-positive count “4” means the system generated only 4 false alarms over the month, a manageable number for real-world deployment.

For congestion detection, the high congestion detection rate of 95.3% (81/(81 + 4) × 100% = 95.3%) confirms that the framework reliably identifies most congestion events, while the low false-alarm rate of 4.7% (4/85 × 100% = 4.7%) demonstrates its ability to distinguish accidents from routine congestion without excessive false alarms.

## 6. Conclusions

In this paper, we investigate traffic conditions at the single-lane level and propose a rectangular grid-based method for traffic state determination. To account for the dynamic propagation of traffic over both spatial grids and time, we develop a three-dimensional Markov model to predict the evolution of traffic flow following an accident.

By extracting real-time vehicle location information from GPS and mobile phone data within a big-data context, our method enables lane-level traffic state prediction based on rectangular grid partitioning, which strikes a favorable balance between spatial resolution and computational efficiency. The results demonstrate improved accuracy in both accident detection and traffic flow prediction.

Moreover, after determining the traffic state of each grid, we construct a traffic state matrix that integrates the states of all grids in both the longitudinal and lateral directions. Combining historical data with real-time observations, we build a Markov model to predict the spatio-temporal distribution of traffic dynamics. The transition probability matrix incorporates three simultaneous influencing factors, i.e., the upstream grid state, the state at the previous time step (*t* − 1), and the historical state from the previous day (*d* − 1), thereby forming a three-dimensional framework that traces traffic propagation in both time and space. The results yield feasible precision and accuracy for traffic state prediction.

Notably, this paper develops a proactive accident detection method that reduces the detection time and mitigates the potential impacts of accidents. Moreover, the proposed method is also valuable for identifying abnormal traffic conditions and monitoring accident situations. With the availability of vehicle GPS data, the method can be readily applied to road traffic management, traffic state recognition, and predictive modeling in intelligent network and big-data environments.

It should be acknowledged that certain limitations remain for future study. For instance, the current Markov model simplifies traffic propagation along the longitudinal direction only and does not account for state transitions between adjacent lanes in the lateral direction due to computational efficiency and data availability. Future work will incorporate the propagation of traffic flow across lanes. In addition, the parameters of the rectangular grid analysis can be further calibrated to accommodate varying traffic conditions. Furthermore, the current validation is limited to a single road segment and a one-month dataset. Future work will extend the evaluation to multiple road segments with diverse traffic conditions, road geometries, and accident types, as well as longer time periods to capture seasonal variations. Beyond these refinements, recent advances in spatio-temporal modeling offer promising directions for upgrading the current framework: the Mambaformer architecture used in MMFlow (Mambaformer + One-step Mean Flow) [45] achieves linear complexity while capturing long-range dependencies, a valuable feature for traffic prediction where both efficiency and long-term accuracy matter, while the adaptive progressive fusion strategy in APT-TP (Adaptive Progressive Transformer-based Trajectory Predictor) [46], which refines predictions through coarse-to-fine trajectory-scene interaction, could inspire a hierarchical extension of our grid-based approach. Although these methods address micro-scale motion prediction rather than macro-scale traffic flow, their core ideas of efficient sequential modeling and progressive feature refinement provide valuable methodological guidance for future work.

## Figures and Tables

**Table 1:** Sensitivity of prediction MAE to weight configuration.

Configuration	Description	τ (Spatial)	φ (Temporal)	ω (Daily Cycle)	MAE
Baseline	-	0.40	0.43	0.17	0.020
Time only	Conventional first-order Markov	0.00	1.00	0.00	0.032
Spatial only	Upstream propagation only	1.00	0.00	0.00	0.045
Daily only	Historical periodic pattern only	0.00	0.00	1.00	0.048
Time + Spatial	No daily-cycle dimension	0.50	0.50	0.00	0.025
Time + Daily	No spatial propagation	0.00	0.50	0.50	0.030
Spatial + Daily	No temporal continuity	0.50	0.00	0.50	0.041
Equal weights	Uniform weighting	0.33	0.33	0.33	0.026

**Table 2:** Baseline methods description.

Methods	Description
Threshold-only	The triggering condition (μ < 0.28 and congestion duration ≥ 30 s without SVC)
SVC (traditional features)	Using only current occupancy and state duration as inputs, without daily/weekly historical features
MLP (3 layers, 4 features)	Multi-layer Perceptron using the same four features as our method

**Table 3:** Comparison of accident detection performance across different methods.

Methods	DR (%)	FAR (%)	MTTD (s)	Precision	Recall	F1-Score
Threshold-only	95.0	45.2	35	0.12	0.95	0.21
SVC (traditional features)	84.0	14.5	50	0.62	0.84	0.71
MLP (3 layers, 4 features)	88.0	9.2	46	0.76	0.88	0.82
Proposed method (Threshold + SVC+ 3D Markov)	92.5	4.2	28	0.86	0.93	0.89

**Table 4:** Confusion matrix for the one-month detection period.

Methods	Predicted: Accident	Predicted: Accident
Actual: Accident	TP = 2	FN = 0
Actual: No Accident	FP = 4	TN = 81

Over the one-month period, a total of 87 events were observed, comprising 2 actual accidents and 85 non-accident congestion events (e.g., peak-hour congestion). Among the 85 non-accident events, 81 were correctly classified as non-accidents (TN=81), while 4 were falsely flagged as accidents (FP=4). The 2 actual accidents were both correctly detected (TP=2,FN=0).

## Data Availability

The data is unavailable due to privacy restrictions.
